# Sub-standard or Sub-legal? Distribution, Pharma Dossiers, and Fake-talk in India

**DOI:** 10.17157/mat.10.3.7279

**Published:** 2023-09-27

**Authors:** Nishpriha Thakur

**Keywords:** India, Pharmaceutical industry, Security, Drug distribution, Regulation

## Abstract

In this article, I look at Indian pharma ‘dossiers’—the bundles of paperwork that testify to pharmaceutical quality and adherence to regulatory standards—and how they illustrate a wider and ongoing shift from a paradigm of drug safety to one of drug security. By examining how dossiers enact and enable claims of ‘quality’, I argue that it is in a drug’s paperwork—rather than its chemical composition—that quality or fake-ness is produced. Based on interviews with Indian traders and officials, and an examination of how their work has changed over time in accordance with the regulatory shift to drug security, I show that in many instances the paperwork has come to be more important than the pill itself. This analysis contests the dominant pharmaco-regulatory notion of fake-ness, which privileges chemical composition above all else. In this way, my analysis of the dossier shows that drug security is itself a powerful form of fake-talk, one that informs the entire market and the conditions of possibility of international commerce today.

## Introduction: Sandeep explains ‘sub-legal’

In early 2020, I had intended to do in-person fieldwork in India to learn what people exporting and distributing pharmaceuticals thought about widespread claims that fake drugs from India were rampant in international markets (Quet 2021; Olliaro et al. 2020; Nayyar et al. 2019; Johnston and Holt 2014; Newton, Green, and Facundo 2010). Unfortunately, the COVID-19 pandemic made it impossible to travel, so I began phoning and using online platforms like WhatsApp to contact traders. I noticed early on that many traders used the World Health Organization (WHO) terminology (see Sirrs, this issue) in speaking of ‘substandard and falsified’ medicines. In one of my phone calls with a trader I will call Sandeep, I asked him about this phrase, as a way of starting a conversation about claims about fake drugs. At that time, Sandeep had been involved in exporting Indian pharmaceuticals for three decades. The moment I mentioned sub-standard medicines he interjected with, ‘Oh, you mean sub-legal?’

I was about to say no and repeat the term ‘sub-standard’, but I paused. Instead, I replied that the term ‘sub-legal’ sounded fascinating and asked him to tell me more about it. He elaborated in Hindi: ‘Sublegal *kah lo ya* underlegal *kah lo, sab ek hi hai*’ [Whether you call it sub-legal or under-legal, it is the same thing]. To illustrate it, he used the example of the common practice of paying a bribe to an official to speed up or simplify a bureaucratic process such as getting a new passport:

You still get your legal passport, even if you paid the bribe to an officer. It is like that, only here [with medicines], it is good-quality drugs. To get your product to markets, sometimes you have to use these ways, and so it is not fully legal. It is ‘sub-legal’.

I was unsure what Sandeep meant by ‘these ways’ and asked him to clarify. His answer was surprising and revealed an aspect of the problem of ‘fake drugs’ on the global market that was new to me. Even if a medicine is manufactured to the required standards, Sandeep explained, sometimes something small might be incorrect in its paperwork. I had heard traders talk about ‘dossiers’, referring to the bundles of paperwork that contain data about a pharmaceutical product’s formulation, its medical properties, the kinds of illnesses and problems it can treat, any health hazard that the pharmaceutical product might pose, its compliance with administrative regulations, and approvals from other regulatory authorities (for example, the National Agency for Food and Administration Control in Nigeria [NAFDAC] or the US Food and Drug Administration [USFDA]) that had reviewed the product and/or plant. Sandeep went on to explain that if a dossier contained a mistake the drug would be considered ‘sub-legal’, but that did not mean the drug itself was faulty:

If the registration of the drug has expired but the drugs are fine, then they are called ‘sub-standard’ by the WHO and other international regulatory bodies. But is there any compromise on the quality? No. It is the same … There are so many small-scale traders in India and they do not have so much money to hire a legal expert or a consultant who would prepare the dossier for a pharmaceutical product. They would instead go to a consultancy firm and get a ready-made dossier at a much cheaper price in order to get the drugs approved. Do you think there would be a problem with the quality of the medicines that he manufactures? That medicine would also work equally fine.

I puzzled over this for some time. How could it be that in practice the considerations applied to determine a drug’s safety and efficacy had little to do with its chemical composition but everything to do with the administrative processes required to take it to the export market? The answer, I realised, lay in the broader context in which the term ‘sub-legal’ came to life.

## From ‘drug safety’ to ‘drug security’

In the 21st century, governments and international bodies such as the World Health Organization (WHO) have shifted from a paradigm of ‘drug safety’ to one of ‘drug security’ (Hornberger 2018; Caduff 2014; Elbe 2018; Quet 2017). In the drug-safety paradigm, pharmaceuticals were regulated by standardising dosages and ingredients and by ‘soft disciplinary measures, such as fines and warnings and regular visits to production plants and dispensaries’, Hornberger writes (2018, 366). This paradigm, she continues, assumed that violations resulted from ‘ignorance rather than criminal intent’ and that ‘regulatory measures can discipline bad practices into best ones’ (Ibid.).

However, this strategy of disciplining exporters and manufacturers so that they could improve and continue their work was gradually displaced as a different set of assumptions came to prevail. Drug regulators, both national and international, as well as the pharmaceutical industry, began to argue that the real problem was criminal activity ‘operating deliberately and deceptively beyond regulation’ (Idem, 367). With this new framework came a whole new set of security organisations, such as the WHO’s collaboration with Interpol (the International Medical Products Anti-Counterfeiting Task Force or IMPACT), and new practices—labelling requirements, packaging requirements, etc.—designed to exclude criminals from the market. This Research Article examines ethnographically how this change was enacted in the early 21st century and how it was interpreted by traders like Sandeep, who observed the changes on the ground, first-hand, and adjusted their own business practices accordingly.

Pharmaceuticals have been of interest to anthropologists over the last few decades, with many focusing on how drugs and their use are shaped by social contexts. One body of work argues that pharmaceuticals are objects with biographies and social lives that begin the moment people engage with them. For example, Van der Geest, Whyte, and Hardon (1996) considered the ‘life cycle’ of drugs, from production, marketing, and prescription, to distribution, purchasing, consumption, and efficacy. Building on this, Hardon and Sanabria (2017) have argued that there is no pure (pharmaceutical) object that precedes its social use and meaning, redefining the notion of ‘efficacy’ as one that is processual, relational, and situated. Both studies show how a drug’s efficacy is shaped by the route of administration, by the accuracy of the diagnosis of the disease for which the drug is to be administered, and by the diversity in the bodies that consume and metabolise it. Science and technology studies scholars have examined pharmaceuticals as materials that are constantly in the process of becoming something else, given their interactions with other materials in the environment. As ‘informed materials’ (Ingold 2011), they may be manufactured in the ‘aseptic space of the laboratory’ (Barry 2005), but once they are out of the laboratory they interact with so many different materials that their efficacy cannot be the same as that in the laboratory.

Taking these steps to redefine the ‘life’ of a pharmaceutical and to interrogate the notion of efficacy even further, my research shows that under the drug-security paradigm neither the drug itself nor its efficacy is the main object of regulation. Rather, I view the dossier as being emblematic of the paradigm, analysing how Indian pharma traders navigate this domain of drug security both made and sustained by its paperwork. India’s intensely heterogeneous drug manufacturing practices and the unique legal frameworks that govern them make it a good site for studying the workings of the drug-security regime at present.

Concerns about the quality of Indian pharma have been raised in relation to a number of high-profile international controversies (particularly in debates about access to medicines) (Mbali 2013; Sunder Rajan 2017). The accusations are not, however, evidence-based (Hodges and Garnett 2020). Even more interestingly, in studies that blame a sub-standard drug for a large number of deaths the methodology typically entails checking the drug’s paperwork but not conducting a chemical analysis of the compound. Thus, it appears that paperwork alone is enough to designate drugs as sub-standard. This is in spite of these same studies acknowledging that it is not possible to accurately judge this using just one form of analysis (Caudron et al. 2008; Nayyar et al. 2019; Almuzaini, Choonara, and Sammons 2013).

My interviews with pharmaceutical traders in India suggest that while dossiers are somewhat like promissory notes, promising quality, even this function is suspect because it is unclear what actually backs up the promises they make. The shift from drug safety to drug security has not only changed regulatory quality-assurance practices, it has also fundamentally changed what is being regulated: ‘drug security’ means that it is no longer the pill itself but its paperwork that matters. I argue that we might better imagine these dossiers as passports, allowing medicines to travel—or not—into markets. Having the financial resources and political clout to produce a better dossier for a given drug means being able to capture a greater share of the market, even if a drug’s chemical composition is identical to one with a more meagre dossier. Thus, this article shows how, in the shift to drug security, the actual pills have faded from view. ‘Quality’ has decoupled from pharmacology, and a well-resourced corporation can easily gain the upper hand in the market. It is not that pharmacological testing ceases or becomes irrelevant. Instead, pharmacological testing is incorporated and subsumed within the dossier. In turn, the dossier takes on a life of its own. In particular, under the drug-security regime it is the dossier that is the primary object of scrutiny. As I will develop, the dossier is also the site upon which fake-ness is declared.

Between March 2020 and June 2021, I conducted interviews with owners of 15 small- or medium-sized pharmaceutical manufacturing companies, five Indian pharma export distributors (i.e., those who connect the exporter with an interested importer), and three drug regulation board officials at the Indian government. I was able to find my participants’ details, such as their mobile numbers or email addresses, from their company websites or from LinkedIn and contacting them directly. Typically, our conversations lasted well over an hour, and I was able to have at least two or three conversations with each trader over the course of a year. In most cases, I was also able to ask for clarification through WhatsApp. In addition, I attended multiple industry webinars^1^ about how to stay compliant with international trade standards, organised by Indian pharmaceutical trade associations.

In my research I focused on small- and medium-sized players because not only do they constitute the majority of pharma businesspeople in India,^2^ they are also much closer to their company’s everyday work of maintaining compliance with trade regulations and its know-how. These companies are usually not large enough to be able to afford their own in-house consultants to produce dossiers and manage all the details on their behalf and will in most cases do it themselves.

Through these interviews, I learnt that the rise in use of the drug ‘dossier’ is a story of the social life of the drug-security paradigm. The traders described to me how they navigate India’s pharmaceutical export markets and negotiate the drug-security paradigm on the ground. Consequently, here I analyse regulation as a thing not in itself but as a processual framework enacted through people doing things. Here, as I go on to show, the dossier becomes a tool for navigating both the market and questions about the quality of pharmaceuticals.

To put it simply, the basic question I asked interviewees was what does a drug inspector actually inspect? In March 2021, once restrictions to fieldwork research lifted in India, I was able to visit a drug inspector in Lucknow. When I arrived, he was busy answering phone call after phone call. Finally, he took a break, and I took the chance to explain that I was curious about how inspections happen and how he determined which drugs are counterfeit, fake, or unauthorised. This is how he replied:

I just go through the dossier. Simple. If I find something wrong with the numbers, such as, let’s say, a stability point of a particular drug at a particular temperature, then I know instantly that there is something wrong with either the drug or the manufacturing facility and I hold off the license.

I was confused, especially by the idea that he didn’t actually go to the plant or do any lab-based pharmacological testing. Perhaps sensing my confusion, he added, ‘I have been in this business for the past two decades. I do not even need to go to the plant, I can tell from the documents’.

One might presume that a drug inspector would inspect *actual drugs*. Instead, what I found is that the rationale behind the drug-security paradigm has remade what drug-quality assurance looks like in practice. In what follows I offer an account of my attempts to understand both Sandeep’s claim and the social life and significance of the term ‘sub-legal,’ as well as its broader, provocative implications for how we understand what claims about fakeness, or fake-talk, actually accomplish (Hornberger and Hodges, this issue).

## From medical representatives to matchmakers

I was able to make sense of my interview with Sandeep—and his curious term, ‘sub-legal’—sometime later, when I viewed it in the broader context of international pharmaceutical manufacturing, trade, and distribution and the changes within it that have transformed the sector since the close of the 20th century. Sandeep’s career trajectory illustrates this transformation. He began working in pharmaceuticals in 1994 upon finishing his studies, which included a postgraduate qualification in pharmacy. He started out as a medical representative in one of India’s largest and oldest generics companies. A medical representative is a personalised marketing agent who promotes a company’s products through financial incentives (e.g., discounts) and in-person visits to physicians to inform them about new medicines.

Sandeep went on to explain that he had been ‘promoted to area [sales] manager just after one year of being an MR [medical representative]’. As such, he came to lead a team of about a dozen medical representatives, each of whom would have an account of around 100 physicians whom they would visit regularly. Sandeep and those on his team would speak with ‘at least 12 doctors every day and go and meet them according to their erratic time schedules’. Sandeep went on to explain how gruelling his first few years in the sector were as he got to ‘know the market’:

To show that I was good in my territory, I would skip meals and rush from one doctor to another. I would learn about the latest medical trends and talk about them to the doctors. I would explain how [the company] was the brand to trust. Sometimes, I would wait in a doctor’s waiting hall for several hours. That actually helped the doctors perceive that I was serious about what I was trying to sell. I had to excel in both—pharmacology as well as trade.

The pharmaceutical business, even in the 1990s, was a complex network of actors that included doctors, wholesalers, medical representatives, pharmacists, and shopkeepers. In an elaborate account of how medicines sales happened in Mumbai in the 1990s, Kamat and Nichter (1998) looked at the prescribing capacity of shopkeepers and the need to regulate pharmaceutical sales. Acting as intermediaries, medical representatives helped pharmacies to liquidate unsold and slow-moving stocks. Kamat and Nichter’s (1998, 784) work also sheds light on why Sandeep himself preferred to become a medical representative over working as a pharmacist: medical representatives with even just a few years’ experience could earn lucrative rewards for meeting their sales targets, such as gold items or holiday packages paid for by the pharmaceutical company. Horner (2014), discussing the growth of the Indian pharmaceutical sector, has also described how medical representatives were able to gather ‘technical knowledge’ and then start their own companies after they had learnt all the ‘new technology’.

Certainly, to be able to navigate the pharmaceutical industry Sandeep needed both an understanding of pharmacological quality and knowledge of the various actors in the industry such as doctors, pharmacists, retailers, and consumers. Moreover,in this ‘symbiotic network’ (Kamat and Nichter 1998) there were implicit market regulations to be aware of: if a customer was not happy with a drug, they would not purchase it again; if a shopkeeper was unable to liquidate the stock, they would not refill an order; if a medical representative was not able to build a rapport with a doctor, they would not be invited to return. Pharmacological quality was in many ways a hinge point between the actors, even if generating profits was the prime motive because it was the only way to survive in the market.

What is significant about Sandeep’s career is that it begins at the tail end of what can be referred to as the ‘drug-safety moment’, when pharma was largely governed by a set of regulations that attempted to standardise drug manufacture as a way of standardising drug quality itself. Who set these drug-safety standards and how were they enforced? The key player was the World Health Organization (WHO) which, in the final decades of the twentieth century, published a series of guidelines and standards that were widely accepted by major pharmaceutical manufacturers. The point of them was to set benchmarks for drug quality by identifying essential ingredients or active pharmaceutical ingredients and stating how much of each should be in a certain medicine. If a drug met these standards it could be deemed effective and safe. Within this drug-safety paradigm, ‘drug quality’ was fundamentally tied to a drug’s pharmacological properties (WHO 1997, 9–12).

Pharmaceutical regulations in most countries set up during the second half of the 20th century stemmed from concern about the ‘quality’ of drugs, where quality was defined by their identity, purity, potency, and uniformity (Sirrs, this issue). The issue of quality first arose in the international drug trade in the 1970s and 1980s, at a time when US and European companies were the key suppliers of pharmaceuticals. Global South countries and regulatory authorities began to complain that medicines being exported to low-income countries were sub-standard and insisted that exporting countries be responsible for ensuring the quality of the drugs they traded. At around this time, concerns that sub-standard drugs were circulating in the markets of Global South also emerged, drawing attention to the role of the national drug regulatory authorities (NDRAs). To ensure that sub-standard drugs did not enter the markets, the NDRAs delegated responsibility to all the actors involved: manufacturers, wholesalers, importers, governments, and even the WHO. In 1969, the WHO set guidelines defining the principles of quality control and laid out general considerations for ‘good manufacturing practice’ (GMP) that included factors such as premises, personnel, equipment, packaging, and labelling. At around the same time, the WHO also developed model certificates (to assure importers of the good quality of the drugs) to be adopted by the NDRAs. It is interesting to note that as the certification scheme was predominantly paperwork, compliance or enforcement was based on trust and regulatory capacity. These model certificates, however, also laid the groundwork for the drug-security paradigm to flourish.

Sandeep joined the sector at a point when having pharmaceutical knowledge—about the pill itself and its contents—was pivotal to the very possibility of trade or sales. But the time of pharmaceutical knowledge’s precedence was soon to pass. Sandeep continued: ‘Nowadays, it is only in trade that people excel. So we have also adapted to that. Now I do not invest so much time in learning about drugs and their usages—something called “continuous medical education”’.

It is worth pausing here to explore what Sandeep means by ‘trade’. Here, it means sales talk based not on the pharmacological properties of a given pill but on a new framework in which the very possibility of trade is based on a company being able to meet regulatory approvals. What is most striking for my purposes is that a given company must secure these approvals far in advance of any actual manufacture of a drug. It is pertinent to note that when Sandeep waited in the doctors’ lobbies, he had a sample of the pills that he wanted to market; later, his work entailed emailing paperwork before any manufacturing began. He explains:

Now my work is mainly to sort data for market viability. I make categories of distributors: 1) who can invest a lot of money and has a good name (credibility) in the market, 2) who can invest but does not have a good name in the market, and 3) who cannot invest but has a good name in the market—and I link them up with appropriate traders. I do market viability research for my clients: what kind of medicines would be in demand in Nigeria, what kind of medicines in Uganda? These demands have to be thought of before buying … But you see, all of this work is now online. No longer is it visits to the doctors, to the medical shops. I can send 100 such emails today, just cut copy and paste, and even if 10 respond, it works great for me. So in that way, the market has changed nowadays.

To understand the significance of Sandeep’s workplace changing from doctors’ waiting halls to his email inbox, of his role changing from medical representative to matchmaker, and of the move from in-person inspections to digital dossiers, one must understand the wider regulatory changes involved in the shift from drug safety to drug security.

## Regulations, patents, and policing

Tracing these significant changes in the 21st century, scholars show how international trade in pharmaceuticals came to be governed by not only a new set of regulations but also a new rationality (Caduff 2014; Chorev 2020; Elbe 2018; Hornberger 2018). These included the emergence of international intellectual property regulations and agreements (e.g., the International Medical Products Anti-Counterfeiting Task Force [IMPACT] and Trade-Related Aspects of Intellectual Property Rights [TRIPS]) and the new importance of patents for pharmaceuticals.

Also key to understanding the emergence of the drug-security paradigm was the new role of international policing in ensuring compliance with a relatively new global intellectual property (IP) regime. IP regulations combined with real-time policing produced new optics for understanding what a pharmaceutical was. For example, the status of a medication’s patent compliance came to stand in for its quality (Hornberger 2018, 368). Drug security also came about as part and parcel of the coincidence of health and national security arrangements in the wake of 9/11 and anthrax scares in the United States (Elbe 2018), a conflation that saw health concerns gain a much higher profile than ever before. Security and health converged with the aim of avoiding and combatting threats to health, not only in the present but in the future too.

From concerns about health security and biosecurity came a new field of threat and risk—both imminent in pharmaceuticals—which itself became the target of new regulation (Caduff 2014). As Caduff (2014) argues, by declaring something a security issue we are declaring that it can never be secure again. Hornberger (2018) concurs, pointing to a dialectics of ‘excess’ and arguing that within a drug-security paradigm the potential threat is unending and infinite. This in turn spills over into and authorises what Masco (2017) calls the ‘militarization of public health’; in his telling, not only is everything ‘not secure’, but everything in global trade also needs constant security and inspection.

In short, what the ‘drug-security paradigm’ refers to is a reorganisation of the bureaucracy governing drug quality. What had started out as the ‘gold standard’ for ensuring drug safety, that is, pharmacological testing, came to be replaced by paperwork and bureaucratic compliance. Of the important changes to international trade regulations at the close of the 20th century, the most noteworthy here is the inclusion of pharmaceuticals in global IP regulations.

## Same drugs plus different dossiers equals different markets and value

Let us now move to the dossier itself, since it is the vehicle by which drugs travel to markets—although, as I will show in this section, different dossiers unlock specific markets. A key document in the making of the dossier as a tool in pharmaceutical trade is the World Health Organization's (WHO) ‘Quality Assurance of Pharmaceuticals: A Compendium of Guidelines and Related Materials’ (WHO 2007), which lists all the documents that manufacturers must show an inspector:

These may include the manufacturing license, the marketing authorization dossiers for [the manufacturer’s] leading products … complaints and recall records, the results of regulatory (surveillance) testing, and the previous inspection reports … Company documents, including the annual report for the shareholders, the complaints file, and self-inspection/internal audit reports. The inspector also should be given information pertaining not only to the particular manufacturer, but to the career of the drug globally such as ‘reports of adverse drug reactions’ (WHO 2007, 295).

For the purposes of my analysis what is notable is that of the 11 items on the list, arguably only one concerns pharmacological quality (‘the results of regulatory (surveillance) testing’). The other 10 are ‘meta data’ about the drug and the company; in other words, information about information.

The dossier described in the WHO guidelines is necessary only if a manufacturer decides to export a drug. Before a dossier can be assembled, the manufacturer must obtain a Certificate for a Pharmaceutical Product (CPP) from the appropriate national regulating body. In India, this is the Central Drugs Standard Control Organisation, which certifies whether or not the drug complies with standards set out in the WHO’s GMP. To trade in importing countries that require compliance only with the WHO’s GMP (e.g., Cambodia), the manufacturer does not need to include any additional information in the dossier about the securitised environment in which the drugs are produced, and sometimes does not even need a dossier. If the importing country has more stringent regulations, however, a dossier is needed and must include additional data as required by the drug regulatory authority; in some cases, it may also require certification of an on-site inspection by the regulating authorities of the importing country. If the manufacturer complies with US Food and Drug Administration (USFDA) requirements, then paperwork confirming that is the only requirement that needs to go into the dossier, since compliance with USFDA requirements is considered globally to be one of the most stringent.

These distinctions reveal how different export markets require different dossiers, even though the drugs involved might be exactly the same. For example, as explained above, some export markets might require only WHO GMP-compliant drugs, whereas others might require something else. In fact, USFDA-approved drugs may actually be manufactured in the same or similar facilities as other drugs that do not have such approvals are—the factory itself does not determine the quality of a drug. This is a common reality in India, due to its peculiar regulatory system that allows for so-called ‘loan licensing’ and ‘third-party manufacturing’ (Rault-Chodankar and Kale 2022). In other words, there is a latticework of production and export stages (Horner and Murphy 2018). For example, one company might import the active pharmaceutical ingredient; another company may buy this ingredient from the first and manufacture the pills; another still may put the pills into blister packs and package them into retail-ready boxes; and so on (Rault-Chodankar 2020).

What determines the difference between the quality of pharmaceuticals then, is the quality of the dossier. Larger players, even those who get their manufacturing done through loan licensing with small-scale industries, can more smoothly navigate the export process because of the quality of their dossiers and because their in-house consultants are fluent in international trade regulations for pharmaceuticals. But how do small-scale traders function in this setting? Let us look at a few examples of how this group uses the dossier as a tool for navigating market access under the drug-security regime.

When I met Surendra, he was exporting from Mumbai while his drugs were being manufactured in the neighbouring state of Gujarat through a ‘third-party manufacturing’ arrangement. He explained that it is the dossier preparation that is the most important element in the process. Having graduated with a bachelor’s degree in commerce, Surendra decided to invest his capital in pharmaceutical exporting in 2004 after consulting his wife, Madri. Madri, a lawyer, works at a pharmaceutical company preparing dossiers for various pharma companies in India that wish to export to any country in the world. Surendra told me that he had no idea about the industry, and when his wife told him that they should invest in and export pharmaceuticals from India he was worried. He recalled telling her that it would be difficult because neither of them was a trained pharmacist, and continued:

But she convinced me, you know. She told me that it is not so much about knowing the medicines and biology and all. It is about knowing the rules, about the country where we are exporting to and its demands, and how to get the plants approved. I decided to trust her. I was not able to find a stable job anyway so decided to give this a try. And she was right, we really found our space in the pharma market.

Thus, the heterogeneity of the Indian pharmaceutical industry allows actors like Surendra to enter the market and prepare a dossier with ‘as much capital one has’ and then ply their way through. The dossier becomes a navigating tool, rather than evidence of pharmacological quality. Quet (2017), who has looked at how distribution alters pharmaceutical value, argues that distribution is often misunderstood as merely a stage of transportation and that scholars should look to the securitisation and authorisation of distribution channels in creating pharmaceutical value. Hence, when Surendra says that it is not so much about ‘medicines and biology and all’ and more ‘about the rules’, one can see how the drug-security paradigm shapes the process of distribution in certain ways that generate pharmaceutical value. Adding to this, he told me,

[Madri] knows that it would not be possible to get your drug registered in Cambodia without paying 100 USD extra to the officials. But at the same time, she also knows how to work with the expiry dates because, you know what, our [Indian] drugs are not expired, they are great in quality but then they [importing countries] want us to pay fines just like that, or officials want extra money so they threaten that if we don’t pay extra, our drugs would be lying in the containers in customs clearance till the expiry date of registration.

The ‘100 USD extra to the officials’ mentioned here is an additional payment required to ‘improve’ the quality of the dossier; that is, it is an additional cost required as part of navigating the export market. In this, I draw a similar analysis to that of Peterson (2014), who has studied the changes in Nigeria’s drug regulatory environment. Looking at which practices feature in reconfigured networks and systems, Peterson (2014, 80) shows that larger companies are able to create end-to-end logistics networks and thereby remove the hassle of paperwork, whereas smaller companies must devote resources to figuring out the paperwork in order to enter the market. Peterson (2014, 84) argues that regulatory processes are used to contest who is entitled to control and potentially accumulate great wealth in large, high-volume, traded markets.

Surendra’s account illustrates how the drug-security paradigm has paved the way for payments for paperwork to become central to ensuring passage into markets. Payments made by manufacturers, distributors, and other kinds of brokers to different kinds of regulatory authorities and channels, at various regulation checkpoints, formalise drugs by facilitating the processing of paperwork that creates legal drugs. The medicines that Madri and Surendra export might be perfectly safe and effective, but if they do not pay the 100 USD fee at the port they risk their registration license (as opposed to the drugs) expiring, thereby rendering their drugs de facto ‘not secure’ regardless of their pharmacological quality.

Inverardi-Ferri (2021, 1653) argues that illicitness is not an ontological category of drugs and that drugs may move in and out of the illegal category; for example, a drug may be illegal in one country but completely legal in another. In contrast to this geographical perspective, it is less about the drugs physically or geographically moving from legal to illegal—manufacturing, distribution, and consumption might all take place in different locations, and so geography it may simply reflect the stage of the process the drug is in. Rather, I argue, illicitness is about drugs being part of a securitised economy, wherein what comes first is the assembling of the paperwork and ensuring its quality. The actual physical material of the drug comes into the picture much later. Surendra’s case shows how the pill recedes from view and is supplanted by its paperwork.

## The vagaries of dossiers

Since securitisation has to happen before the drugs are even manufactured, so the dossier comes into being before the drugs are manufactured. Let us look at how that happens through the experience of another small-scale trader. Amog, from Mumbai, exports paracetamol to East African countries. Amog studied pharmacy at Mumbai’s leading college and takes pride in being a pharmacist. Throughout our conversation, he kept mentioning that in the last two decades most of the pharmaceutical export business has been taken over by businessmen who are not experts in pharmacological quality and are only interested in making money:

You look at my paracetamol tablets, they even go to Congo, though I don’t have my drug registered there but secretly through some distributors. You know why? Because my medicines work. I have only focused on that from the beginning—create a brand through quality. I know that when my tablet consignments are stuck in customs on the port, they would lose their API [active pharmaceutical ingredients] gradually which is why I add 2% extra—i.e., 500 mg tablet contains 510 mg. When it reaches the patient, it is still effective and so the patient looks for my brand, always. How does it matter if I don’t have large offices like Gujaratis, and consultants and managers sitting in the office?

Even though Amog has confidence in his product, he hired a private consultant, someone like Madri, who would not charge him much for the dossier but could still get his products registered in countries he wants to export to. His market is quite small and he does not make much profit, but he is able to export his products because he was able to find a ‘ready-made dossier’ for paracetamol and could substitute its data for that on his own product. He laments that those who own larger firms are able to employ their own consultants, thereby standing a better chance of getting their plants and products registered in various countries.

Darshanbhai is exactly the kind of person Amog is talking about. Darshanbhai already has a US Food and Drug Administration (USFDA) approved plant and told me that every product he makes is traceable: each product has a QR code that gives its manufacturing conditions. He tells me that he has spent an enormous amount of money on this manufacturing plant, taking care of all the technical upgrading in order for it to match and comply with regulations for global drug compliance. He manufactures sex hormones—progesterone, oestrogen, contraceptive pills, abortion pills, pregnancy-sustaining pills—and tells me that the biggest market for this is in Africa. But he has not been able to get contracts from donors or non-governmental organisations (NGOs) for the distribution of these medicines. He was particularly angry about one instance, in August 2020, when he went to an East African country (he did not want to disclose the name) to meet the main officials there in order to get a contract from an NGO. Unfortunately for him, at the same time he applied for registration Pfizer also applied and duly got the contract. Even though Darshanbhai’s plant was USFDA-approved, he could not secure the deal because, he told me, drug markets are always dominated by the big companies that actually made the regulations. In any case, they are considered to be the best, he tells me, because of this that many Indian manufacturers are not able to get deals from donors and NGOs in Africa. When I pressed him, asking whether Pfizer might indeed have better manufacturing conditions than other Indian plants, he responded:

But who cares about the quality? Pfizer also manufactures in India and has same manufacturing plant. Sometimes, in fact, they do not even manufacture because of loan-licensing facility and third-party manufacturing facility. You can read on their packet that it is *marketed* by this so-and-so brand and in the bottom, *manufactured* at a different place. So if the manufacturing has been done at a different *local* place then the money is only for marketing, isn’t it? It is all about how well you know these officials. Now you know, even WHO Geneva GMP [good manufacturing practice] is different than WHO India GMP. There is no one standard that would work all over the world. You just have to know where your product will sell and try to have the best plant according to the rules of that country.

Even though Darshanbhai could not get the NGO contract in East Africa, he proudly concluded: ‘But so what? I now have a manufacturing plant in New Jersey, USA, and that too is USFDA-approved. If I do not get these smaller markets, I will move to larger markets for selling my drugs’.

This shift of Indian drugs to larger markets like the US, Europe, and Japan is discussed by Chorev (2020). She shows that each year between 2001 and 2010, the number of drugs that were introduced to East African markets from India continued to increase owing to the introduction of drugs like antiretrovirals (ARVs), antimalarial drugs and anti-tuberculosis drugs. Interestingly, the reputed drug companies from India that stayed in the East African markets were already present through contracts with the US President’s Emergency Plan for AIDS Relief (PEPFAR) and the Global Fund. In contrast, those Indian firms who had not been able to secure contracts started to make a shift towards the markets in the Global North, in order to make profit through selling essential drugs in bulk. Hence, the interest of these companies in the East African market may be dependent on their sales to donors such as UNICEF and the Bill and Melinda Gates Foundation, and she suggests that if donors left these markets, the manufacturers would follow (Chorev 2020, 33). To go back to the case of Darshanbhai, though his manufacturing plant has received FDA approval, something that Surendra and Amog continue to aspire to, he still could not secure a deal in East Africa. He hopes instead that his manufacturing plant’s ‘quality’ of being USFDA approved will allow him to move to larger (and more profitable) markets.

In all the cases discussed here, the dossier containing details about pharmacological values about a given drug stands in for the pharmacological quality of said drug. It is the dossier—and not the drug itself—that has to be approved by the regulatory authorities, often ahead of the drug beginning to be manufactured. In a way, then, the quality of the drug is performed by the quality of the dossier. People such as Amog, Darshanbhai, and Surendra are the traders that Sandeep puts into the charts in his market-viability research matching distributors and manufacturer to countries. In a way, Sandeep focuses more on whose dossier would be viable at what level, and then arranges and matches them with distributors accordingly. Even though some traders and drug regulators in my fieldwork claimed that the dossier ‘shows’ a drug’s quality, Darshanbhai’s case questions or rather problematises exactly that assertion. Even if his dossier supposedly had practically the same data that Pfizer’s had, because of the loan-licensing facility Pfizer still got the deal. The more approvals (of different regulatory bodies from different countries) a dossier lists, the easier it seems to become to get further approvals. It would appear that a dossier, rather than a drug, of average quality, is a risky object in the pharmaceutical market.

I also wish to bring in the idea of ‘risk’ from the perspective of economic geography, which asserts that certain regions, particularly the Global South, are considered to have ‘dodgy people doing dodgy things with dodgy goods in dodgy places’ (Gregson and Crang 2017). The drug-security paradigm in the case of India has taken on particular intensity because of assumptions that India does not have a robust regulatory system or that its pharmaceuticals are ‘riskier’ because they do not undergo similar stringent regulatory, or rather paperwork, approvals. In a way then, here the dossier has to outdo itself. While it stands in for the quality of drugs, it can never be enough.

## Conclusion: Drug security as fake-talk

The conditions of possibility for the drug-security paradigm have been generated by international actors such as the World Health Organization (WHO), the research and development-based pharmaceutical industry, and the national drug regulatory agencies (NDRAs), all of which have voiced the concern that global health is under threat from criminals’ intents on trading in fake medications. Once criminality is associated with an object (for instance a drug) it is easy to associate it with different kinds of risks and to securitise not just its distribution but also its manufacture. The life of a drug begins even before its manufacture—right when the proposition to make the drug happens in a dossier —and this both produces and is produced by an infinitely expansive understanding of risk and threat that is part and parcel of what drug security seeks to securitise and the bureaucratic process that derives in drug manufacturing approvals.

The network of specific moment in the social life of drug—dossier writers, traders, manufacturers, inspectors—does not chemically alter the drug but nevertheless changes perceptions of its quality. Banerjee’s (2016, 376) has argued that hyper-technical language used by global pharmaceutical companies

‘configures molecular life, the global market and legal language such that corporations emerge as the true upholders of international law and the sole gatekeepers of legitimate pharmacogenomics and any entity that challenges this appears to be archaic, contractually illegitimate and producers of dubious biochemical quality’.

Paperwork changes perceptions about quality. The dossier also enacts the quality of the drug and is constantly evaluated in the drug-security paradigm. By examining the dossier closely, we can appreciate how the very bona fides of drug quality begin life not in the manufacturing process of active pharmaceutical ingredients nor in their formulation, nor even in their packaging, but in the documentation that must be approved by drug inspectors prior to any object even being produced. The common chronological understanding of how a drug’s quality is assessed is also ruptured, because a drug’s quality is produced not in the manufacturing stage, nor through distribution or consumption: it is declared long before a drug is even manufactured. We could say that quality remains not in the pill, but in the dossier.

## Footnotes

1 These included: ‘The Importance and Impact of Smart Packaging on Pharma Industry’, organised by the Indian Drugs Manufacturers’ Association (IDMA), 2 April 2021 on Zoom; ‘Global Markets for Pharmaceutical Exports’, organised by the IDMA, 13 March 2021 on Zoom; and ‘Issues, Challenges and Opportunities of Pharmaceutical Industry: India Perspective’, organised by the IDMA and the Gujarat Pharmaceutical Association, 21 November 2020 on Zoom.

2 See https://niti.gov.in/planningcommission.gov.in/docs/aboutus/committee/wrkgrp12/wg_pharma2902.pdf.
